# Oxidative Stress in the Newborn

**DOI:** 10.1155/2017/1094247

**Published:** 2017-01-31

**Authors:** Giuseppe Buonocore, Serafina Perrone, Maria Luisa Tataranno

**Affiliations:** ^1^Department of Molecular and Developmental Medicine, University of Siena, Siena, Italy; ^2^Department of Neonatology, Wilhelmina Children's Hospital, University Medical Center, Utrecht, Netherlands

Oxidative stress (OS) occurs when there is an unbalance between free radicals (FR) production and antioxidant capacity. OS can be a risk factor for fetal programming, representing a key process linking adverse fetal growth, impaired fetal well-being or preterm birth, and later increased risks of diseases in adolescence and adulthood. Adverse outcome to the offspring can extend beyond the neonatal period and includes neurodevelopmental disorders (motor and cognitive problems, attention deficit hyperactivity, and psychotic disorders), asthma, insulin resistance, diabetes mellitus, hypertension, coronary heart disease, and stroke. Free radicals can alter gene expression or damage lipids, proteins, and DNA at a critical developmental point leading to a higher susceptibility to many disorders.

The present special issue aims to stimulate the continuing efforts to understand the pathophysiology underlying OS damage in neonates. Furthermore we aimed to highlight new possible strategies to treat or prevent OS-mediated diseases and to evaluate neonatal outcomes.

This issue includes papers on (i) recent developments in OS-mediated diseases in fetus and newborns in both human and animal models; (ii) advances in identification of prenatal and neonatal characteristics of the infant/fetus at increased risk of OS-mediated damage; (iii) mechanisms of OS-mediated tissue damage using model systems; (iv) recent advances in antioxidant and other protective strategies.

Each manuscript was reviewed by at least 2 external reviewers and one guest editor. We received about 20 papers and only the present 8 papers were selected for the final draft. All the papers were selected on the basis of relevance and novelty for the reader.

The review by Y. Ozsurekci and K. Aykac is an overview on the complex and wide world of oxidative stress pathophysiology and its link to neonatal diseases.

M. Chełchowska and colleagues examined the relationship between selected adipokines and markers of oxidative stress/antioxidant defense in the umbilical cord of neonates exposed and nonexposed in utero to tobacco smoke. Maternal smoking is considered as a source of oxidative stress, which has been implicated to disrupted adipokines expression in adipose tissue. To support this thesis, they found that cord serum visfatin, oxidized low density lipoproteins, and total oxidant capacity were significantly higher, while adiponectin and total antioxidant capacity were lower in smoking group than in nonsmoking group, demonstrating that maternal smoke enhances oxidative status and depletes antioxidant potential in otherwise “healthy” term newborns.

The research group from Italy showed, in the paper authored by S. Perrone et al., a relationship between pain degree and OS in healthy full-term newborns. They found that the amount of OS is gender related, being higher in males. Furthermore nonpharmacological approaches were able to reduce pain score together with pain-related OS in healthy term newborns.

The previous paper links to the paper by J. Wilhelm and colleagues, with the first focused on term healthy newborns and the latter on the effect of OS on fetuses and developing rat brains. In this study authors were able to localize reactive oxygen and nitrogen species (RONS) production in the developing rat brain. In particular, the fetuses showed moderate RONS production, which changed cyclically during further development. The periods and sites of peak production of individual RONS differed, suggesting independent generation. They showed dramatic changes in the amount and the sites of RONS production on day 4 while the adult animals did not produce increased OS markers.

The subsequent topic highlighted in this issue is centered on OS after heart surgery in the neonatal population. Heart surgery is associated with increased inflammation and the production of reactive oxygen species. The use of the extracorporeal cardiopulmonary bypass during correction of congenital heart defects generates reactive oxygen species through different pathways. They underlined the fact that the immature myocardium is more vulnerable to reactive oxygen species because of developmental differences compared to the adult heart but also because of associated congenital heart diseases that can deplete its antioxidant reserve, thus the importance of preventative strategies such as exogenous antioxidants, use of steroids, cardioplegia, blood prime strategies, or miniaturisation of the cardiopulmonary bypass circuit.

D. Hoeber and collaborators made an interesting overview on cerebral white and grey matter injury as the leading cause of an adverse neurodevelopmental outcome in prematurely born infants and on the role of erythropoietin as a promising antioxidant drug. They focused on motor-cognitive outcome up to the adolescent and adult age in an experimental rat model of preterm brain injury. Oligodendrocyte degeneration, myelination, and modulation of synaptic plasticity-related molecules were evaluated in rats exposed to hyperoxia. The analysis of white matter structures revealed a reduction of acute oligodendrocyte degeneration in erythropoietin treated animals. A single erythropoietin administration reverted hyperoxia-induced reduction of neuronal plasticity-related mRNA expression up to four months after injury, highlighting the importance of erythropoietin as a neuroregenerative treatment option in neonatal brain injury.

An intriguing research coming from United Kingdom, authored by E. Rocha-Ferreira and colleagues, deals with protective strategies against OS after asphyxia. The authors found that immediate remote ischemic postconditioning reduced brain nitrotyrosine formation in a piglet asphyxia model. Thus remote ischemic postconditioning (RIPostC) is a promising therapeutic intervention that could be administered as an alternative to cooling in cases of perinatal hypoxia-ischemia. The RIPostC beneficial effect seemed to be mediated by modulation of nitrosative stress, despite glial activation.

Another paper, by S. Manti and colleagues, focuses on causes of atopy. Extensive epidemiological and laboratory studies have been performed to identify the environmental and immunological causes of atopy. Till now, genetic predisposition seemed to be the biggest risk factor for allergic diseases. However recent findings suggest that the establishment of a peculiar epigenetic pattern may also be generated by OS and perpetuated by the activation of OS-related genes. The paper is centered on analyzing the role of maternal and neonatal oxidative stress and oxidative stress inducible genes in order to review the current knowledge about the relationship between maternal and neonatal OS-related genes and the development of atopic diseases.

The wide spectrum of review and research articles presented in this issue provides new inputs and new insight into the pathophysiology, diagnosis, and treatment of OS injury in neonates both for clinical practice and for neonatal research. We call for further investigation on OS in the perinatal period in order to find new tailored strategies to treat or prevent OS-mediated diseases, with the aim of ameliorating neonatal outcomes.

*Giuseppe Buonocore* *Serafina Perrone* *Maria Luisa Tataranno*

